# Data-Driven Performance Evaluation Framework for Multi-Modal Public Transport Systems

**DOI:** 10.3390/s22010017

**Published:** 2021-12-21

**Authors:** Ana Belén Rodríguez González, Juan José Vinagre Díaz, Mark R. Wilby, Rubén Fernández Pozo

**Affiliations:** Group Biometry, Biosignals, Security, and Smart Mobility, Departamento de Matemática Aplicada a las Tecnologías de la Información y las Comunicaciones, Escuela Técnica Superior de Ingenieros de Telecomunicación, Universidad Politécnica de Madrid, Avenida Complutense 30, 28040 Madrid, Spain; anabelen.rodriguez@upm.es (A.B.R.G.); mark.wilby@upm.es (M.R.W.); ruben.fernandez@upm.es (R.F.P.)

**Keywords:** public transportation systems, multi-modal mobility, smart card data, origin–destination matrix, entry-only automatic fare collection systems

## Abstract

Transport agencies require accurate and updated information about public transport systems for the optimal decision-making processes regarding design and operation. In addition to assessing topology and service components, users’ behaviors must be considered. To this end, a data-driven performance evaluation based on passengers’ actual routes is key. Automatic fare collection platforms provide meaningful smart card data (SCD), but these are incomplete when gathered by entry-only systems. To obtain origin–destination (OD) matrices, we must manage complete journeys. In this paper, we use an adapted trip chaining method to reconstruct incomplete multi-modal journeys by finding spatial similarities between the outbound and inbound routes of the same user. From this dataset, we develop a performance evaluation framework that provides novel metrics and visualization utilities. First, we generate a space-time characterization of the overall operation of transport networks. Second, we supply enhanced OD matrices showing mobility patterns between zones and average traversed distances, travel times, and operation speeds, which model the real efficacy of the public transport system. We applied this framework to the Comunidad de Madrid (Spain), using 4 months’ worth of real SCD, showing its potential to generate meaningful information about the performance of multi-modal public transport systems.

## 1. Introduction

The design and operation of a public transport system involves high levels of complexity as it must convey a wide range of perspectives involving, among others, economic investments, service provision, and land use. In their decision-making processes, transport agencies require precise information about the system they manage. Thus, a formal and systematic evaluation is the key to producing optimal outcomes and provide high-quality services [1].

A common way of approaching the evaluation of public transport systems is assessing their topologies and service. These methods focus on detecting the accessibility and vulnerability of the network itself. The former measures whether the users’ are able to reach their destinations on public transport [2]; the latter represents the ability of the transport system to recover from incidents or disturbances [3]. These metrics study the static (topology) or semi-static (operating frequencies) components of the transport system and provide meaningful information about its performance. However, a complete knowledge still requires an analysis of the specific routes traveled by users and the subsequent performance metrics.

The traditional approaches to cover this issue are fundamentally based on simulations and surveys. Simulations are inherently deterministic, which means that, although they are capable of reproducing well-known transport dynamics, they fail to reproduce spurious effects that often carry the most meaningful information about the travelers’ behaviors and the systems’ reactions to them. On the other hand, surveys are incomplete by nature as they must be limited to a predetermined number of people and biased to specific demographic groups (for example, elderly people are hardly represented as they tend to show low response rates). In addition to this, transport behaviors continuously evolve over time; however, given the economic costs, time and effort associated with surveys, they are rarely conducted, which results in huge gaps with no updated knowledge about transport systems.

Although this set of challenges are evidently demanding, the disruption of automatic fare collection (AFC) systems has opened new and innovative approaches to tackle them. Smart cards were originally thought as a simple, agile, efficient, and accurate method of payment. In addition, they provided a higher flexibility with regard to tariffs, improved user experience by reducing waiting times, and decreased the workload on public transport staff. Despite these useful features, their actual potential lies in exploiting these data to provide public transit agencies with valuable information, intensive in space and time, and particularized for individual passengers.

In order to characterize the performance of transport systems, we need to extract the origin and destination of the users’ journeys. These data are directly recorded by some AFC systems such as San Francisco and London, which require exit tapping as tariffs based on the actual traveled distance. However, the majority of AFC systems only register users entering the transport mode (entry-only systems) [4]. This is the reason why the research on AFC systems and origin–destination (OD) matrices estimation has been particularly active over the last two decades. The classical approach to this problem consists of linking journeys following the trip chaining model [5], a simple and effective way of creating an OD dataset that represents travelers’ behaviors in real public transport systems.

We will adopt this same approach, including an extension to it that incorporates specific features of multi-modal mobility, to exploit the spatial similarity observed in outbound and inbound journeys of the same passenger. Using the resulting individual routes, we are capable of constructing a data-driven framework for the performance analysis of public transport systems from a multi-modal perspective.

The remainder of the paper is structured as follows: Section 2 includes a review of the scientific works that study the performance analysis of transport systems from real OD matrices; Section 3 defines a multi-modal journey as the basic information to construct the performance evaluation framework and presents the methodology we followed to create the dataset of individual OD matrices; Section 4 presents the performance evaluation framework and the set of performance metrics it encompasses; and Section 5 discusses relevant issues associated with the calculations and indicates the further research to be carried out in this field.

## 2. Related Work

The fundamental objective of a public transport system is moving passengers between two points in space. As a direct consequence, transport agencies have always been interested in their accessibility as a metric of their ability to achieve this goal, and subsequently the scientific community has produced numerous contributions in this field. One illustrative example of the resulting technologies is the Spatial Network Analysis for Multimodal Urban Transport Systems (SNAMUTS) [6], a GIS-based tool to help agencies in planning their transport networks considering land-use activities. This instrument has been widely used to evaluate public transport systems worldwide as in [7]. Recent approaches extended this methodology to incorporate the concept of level of service and study the correlation between public transport accessibility and education opportunities [8].

Closely linked to accessibility, a second fundamental metric in the assessment of public transport systems is the vulnerability. In this case, not only the network topology must be considered but also the service it provides, analyzing critical links that would generate massive impacts on performance [9] and their associated risk [10]. This type of study generate significant knowledge about specific network design components such as circular lines and how they contribute to reduce vulnerability [11].

In addition to these two basic approaches, performance evaluation can also consider a set of variables in an integrated way. An illustrative example of these multi-criteria approaches is the Analytic Hierarchy Process (AHP) [12], which capable of assessing efficiency, economic costs, and sustainability from a joint perspective. In the majority of cases, these criteria are not equally significant. Consequently, in order to assign specific weights to each of them, scientific works use importance matrices [13] or rankings of key quality indicators [14], among others.

Despite the meaningful information these evaluation tools provide, they mainly adopt the perspective of the network, not considering the actual travelers’ behavior and journeys. This complementary analysis must be inherently data driven, which first requires real individual journeys from origin to destination as the input dataset. However, entry-only AFCs cannot provide this information in a direct way, and thus we need to infer it. This is a classical mobility problem that affects not only public transport systems but also road traffic [15].

There are three main models to deduce the destination of a route: (i) the *trip chaining* model [5]; (ii) the *probability* model, appropriate for inferring the total number of people exiting a station, but not able to provide information regarding each individual passenger [16]; and (iii) the *deep learning* model, only applicable to entry–exit AFC systems [17].

The trip chaining method is based on two assumptions: (1) the traveler starts the next journey at the destination of the previous one; and (2) the traveler ends the last journey of the day at the same station where the first journey started. This algorithm was validated using subway data provided by New York City Transit’s AFC entry-only system (MetroCard), confirming that the starting assumptions were valid for 90% of the users. This work was extended in [18] in order to include multiple modes of transport: subway, bus, ferry, and streetcar, in the MTA New York City Transit (NYCT). Based on this original method, other scientific works have proposed adaptations to fit specific applications, as in [19].

The trip chaining method has been tested on different scenarios. We proceed to review the most relevant studies in this field; an exhaustive list can be visited in [20] and more recently in [21]. This method was tested in Chicago, USA, with bus [22] and subway [23] data. The authors of [24] employed bus data from the city of Gatineau, Canada, where the smart card coexists with payment in cash, estimating two thirds of the actual journeys. In [25], with bus data in Minneapolis–Saint Paul, Minnesota, the study includes a sensitivity analysis of the parameters of the model. The work in [26] uses validations on buses in Jinan, China, correctly estimating 85% of OD pairs during peak hours. OD matrices were also obtained in [27] for buses in London, UK. Data from bus and subway in Santiago de Chile, Chile, were used in [28] in order to estimate 80% of transaction at the exit of the transport systems. In [29], the authors analyze bus and subway data from Rennes, France, using a Gaussian mixture model to classify passengers depending on their temporal profiles; this study obtained an interesting finding: commuters showed a small variance during the morning peak and much larger on the afternoon peak. Finally, in [30] authors inferred destinations for 62% of transactions from bus data in Porto, Portugal.

All the above listed works process data gathered in entry-only systems. Consequently, the validation of the proposed methods, whenever it is available, is based on small sampling personal surveys. However, the trip chaining method has also been tested in systems that provide validation at the exits. In [31], the authors identify multi-modal journeys (on bus, entry only, and Underground gate, both entry and exit) using “Oyster” SCD in London, UK; this research focused on determining thresholds for the transfer times between modes. We can find a second example in [32], which used data from buses, trains, and ferries in South-East Queensland, Australia; in this study, the authors estimated only 66% of the journeys correctly, based on their initial assumption to infer the last destination of the day. Zhao et al. [33] analyzed SCD in Shenzhen, China, a city with 2.8 million daily passengers. Although the card accepted two transport modes (subway and bus), the authors focused their analysis on subway data that included entry and exit validations. They derived spatio-temporal patterns in journeys in order to detect regularity and anomalies. They used statistical methods and clustering techniques to characterize journeys and classify passengers. Thus, they indicate that a high percentage of riders (80%) are spatio-temporally regular, i.e., they travel from the same origin to the same destination at relatively fixed hours. In other words, *riders* are usually *commuters*.

Given the previous analysis of the related work, we can state that our paper provides the following original contributions:

- We develop a performance evaluation framework for multi-modal public transport systems.
- We define and calculate a *new* performance metric, the operating speed, to characterize multi-modal public transport systems.
- We obtain *enhanced* OD matrices that extend the traditional knowledge regarding the percentage of trips between OD pairs, providing meaningful metrics of distance, time, and velocity for these journeys.
- We apply this performance evaluation framework to a *complete* public transport system, including every transport mode.
- We propose an adaption of the trip chaining method to multi-modal journeys to infer OD matrices in entry-only AFC systems.

## 3. The Adapted Trip Chaining Method

### 3.1. Preamble

**Figure 1:**
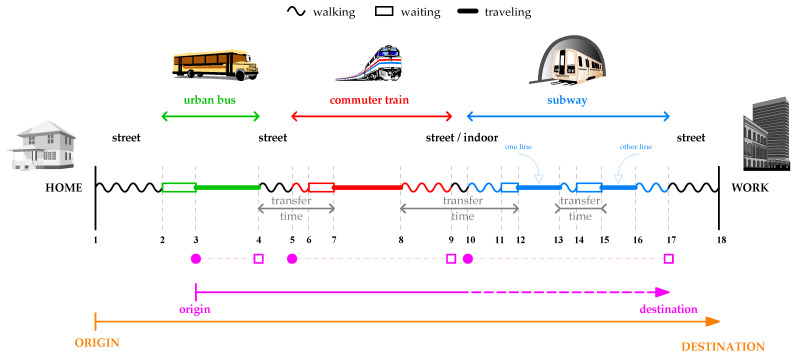
Schematic drawing of a complete journey.

As input to the performance evaluation framework, we need to collect data about the individual routes of users. However, a simple journey is a routine that encompasses a higher complexity than expected. Let us use an explanatory example to detail this complexity. Figure 1 shows a schematic of a complete journey, where the numbered vertical lines represent its relevant items such that the *n*-th item occurs in time $t_{n}$ and in spatial coordinates given by vector $\vec{p_{n}}$.

For simplicity, let us imagine a female traveler who is completing a morning journey from home to her office building. She leaves home in $t_{1}$ and walks to a bus stop, reaching it in $t_{2}$. She waits there until the bus arrives, boards and validates her travel card in $t_{3}$. After a certain time, she alights in $t_{4}$ and walks again to a commuter train station; she validates again on the turnstile in $t_{5}$ and reaches the platform in $t_{6}$. There, she waits until the train arrives in $t_{7}$, boards, and travels until she gets off in $t_{8}$. Then, she exits the commuter train network in $t_{9}$, transits through some corridors, and enters the subway network where she validates her card again in $t_{10}$. She reaches the platform in $t_{11}$, boards the train in $t_{12}$, and alights in $t_{13}$. Still in the subway network, she then changes to a new line reaching its platform in $t_{14}$. There, she waits until the train arrives in $t_{15}$, boards, and travels until she alights in $t_{16}$. She transits through corridors until she exits the subway network in $t_{17}$. She walks some distance and finally reaches her work place in $t_{18}$.

This journey produces 3 validations in total (items 3, 5, and 10) marked in filled magenta points (•) in Figure 1. This is the only available information about the journey provided by an entry-only AFC system, given that it *does not* record validations on the exits (items 4, 9, and 17 represented by empty boxes with magenta outlines (□) in Figure 1). Consequently, although the journey comprises 18 relevant items, we can only observe 3 of them In the SCD, being unable to receive direct information about the remaining 83.3%.

From the point of view of *urban mobility*, this traveler completes the journey represented by the continuous orange line drawn on the lower part of Figure 1, from ORIGIN to DESTINATION: she leaves in $t_{1}$, invests a total time given by $t_{18}-t_{1}$, and travels a distance, following a straight line, given by $d\left(\vec{p_{1}},\vec{p_{18}}\right)$.

The question is: Can we reproduce this journey from the SCD? Is it possible to characterize the *exact* journey, obtaining accurate values for every $\langle \vec{p_{n}},t_{n}\rangle$ pair? Unfortunately, the answer is “no”. Firstly, everything that happens outside the transport system is undetectable for the AFC technology. The walking stretches at the beginning and end of the journey are unknown, and there is no way to determine the values of $\langle \vec{p_{1}},t_{1}\rangle$ and $\langle \vec{p_{18}},t_{18}\rangle$. In fact, although we have supposed that the traveler completed these stretches by foot, they could have also been covered by car. In addition, given that the first transport mode is the bus in this specific example, we could not know the first waiting time, at the bus stop, as we cannot obtain the value of $\langle \vec{p_{2}},t_{2}\rangle$. In summary, there will *always* be a lack of information at the beginning and end of the journey. These stretches that fall out of the transport network cannot be characterized by an AFC system. On the plus side, we can describe the rest of the journey through estimation.

As we can observe in Figure 1, the time that a traveler invests *inside* the transport network can be:

- *Walking time*: Transits made by foot through indoor corridors and streets traveled to (i) reach the boarding platform (which does not apply to the case of buses); (ii) change from one line to another on the same transport mode; or (iii) change from one mode to another.
- *Waiting time*: Since the moment the traveler reaches the boarding point (stop, platform, etc.) until she gets on the vehicle (bus, train, etc.).
- *In-vehicle traveling time*: The time the rider travels on the vehicles, including the times at each intermediate stop.

In the case of buses, walking and waiting times always occur on streets, which makes their estimation even more difficult and impossible at the beginning of the journey.

The time e1lapsed from the end of a traveling period and the start of the next one is called the *transfer time* (see Figure 1). Transfer times always involve some walking (outdoor or indoor) and waiting times. The length of these intervals is highly diverse, depending on the structure of the network, the size and design of stations, and the regularity of the services in each mode, plus some random component related to the time of arrival to the transport system.

Chaining trips imply two main tasks: first, deciding which validations belong to the same journey; second, estimating its unknown destination. A particular research work is then identified by the approach it uses to face these two tasks. Among the methods that are widely employed, we can mention the use of (i) time thresholds to aggregate validations [31]; (ii) spatial thresholds to estimate the destination [24]; (iii) statistical averages to characterize transfer times [28]; and (iv) specific algorithms, such as the schedule-based shortest path algorithm, to determine the most probable path inside a transport mode [5]. In this paper, we will adapt the trip chaining method to estimate the pair $\langle \vec{p_{17}},t_{17}\rangle$ in Figure 1, being able to “complete” the journey drawn as a magenta line between origin  and destination, inferring its a priori unknown part (discontinuous magenta line). In this case, we focus on multi-modal mobility, and thus we will *pair* incomplete journeys based on the spatial similarity of routes traveled by the same rider in opposite directions.

### 3.2. Methodology

Within this work, we define a *journey* as a movement in a specific direction (outbound or return), from an activity to the next, with a particular purpose. A journey is composed by a set of segments that we name *trips*, defined by transfers that imply a validation within the same transport mode (from one bus to another) or among two different modes (from subway to bus, for example). The SCD recorded by an entry-only AFC system during a journey allow us to know the origin and intermediate stations, but not the destination (in which there is no validation).

Let $V_{k,d}$ be the time ordered set of the $N_{S}$ validations made with a smart card *k* on day *d*. Each validation is associated with a spatial location $\vec{p_{n}}$ (including the latitude and longitude of the station where the validation was recorded) and a time stamp $t_{n}$:

$$V_{k,d}={\left\{\langle \vec{p_{n}},t_{n}\rangle \right\}}_{n=1}^{N_{S}},\;t_{1}<t_{2}<\cdots <t_{N_{S}}.$$

We group the validations in sets considering the time interval between each pair of consecutive validations. Specifically, fixing a time threshold δT, if $t_{n+1}-t_{n}\leq \delta T$, the two corresponding validations will belong to the same subset, and if $t_{n+1}-t_{n}>\delta T$, they will be separated in two different subsets. We will use the following numbering for validations within each subset:

$$\left(1,\_\_\_,N_{1}\right),\left(N_{1}+1,\_\_\_,N_{2}\right)\cdots \left(N_{S-1}+1,\_\_\_,N_{S}\right).$$

Consequently, we have a partition of the set $V_{k,d}$ defined as:

$$V_{k,d}={\left\{{\left\{\langle \vec{p_{n}},t_{n}\rangle \right\}}_{n=N_{s-1}+1}^{N_{s}}\right\}}_{s=1}^{S}$$

satisfying:

$$\begin{array}{cc} \; & \left(i\right)\;t_{N_{s-1}+i+1}-t_{N_{s-1}+i}\leq \delta T:\\ \; & \;\;\;\;s\in \left[1,S\right],\;i\in \left[1,N_{s}-N_{s-1}-1\right]\\ \; & \left(\mathrm{ii}\right)\;t_{N_{s+1}}-t_{N_{s}}>\delta T:\\ \; & \;\;\;\;s\in [1,S-1] \end{array}$$

with $N_{0}=0$ and *S* the number of created subsets.

Each element in the partition,

$$T_{k,d}^{s}={\left\{\langle \vec{p_{n}},t_{n}\rangle \right\}}_{n=N_{s-1}+1}^{N_{s}},$$

is a subset of validations that we call *stretch*. Two consecutive validations in this subset delimit a *trip*; the time difference between both of them is the travel time of the trip, which accounts for the sum of the walking, waiting, and traveling times. A stretch represents a *non-ended* journey because its last station (where the traveler left the public transport network) is always unknown given the lack of validations at this exit. Note that there are $N_{s}-N_{s-1}$ validations and $N_{s}-N_{s-1}-1$ trips in a stretch.

From this point on, we will relax the notation for the sake of clarity. Let $T_{k,d}^{i}$ and $T_{k,d}^{j}$ be two stretches with the same number of validations, i.e.,

$$N_{i}-N_{i-1}=N_{j}-N_{j-1}=T.$$

Let us consider the sequence of spatial locations that define each stretch, plus one last unknown location (the destination of the journey). For the first stretch, we have:
$\vec{a_{1}}$⟶$\vec{a_{2}}$⟶⋯⟶$\vec{a_{T}}$⤏?
and for the second:
$\vec{b_{1}}$⟶$\vec{b_{2}}$⟶⋯⟶$\vec{b_{T}}$⤏?

The adapted trip chaining method couples, in inverse order, two incomplete journeys whose intermediate stations match, satisfying some *spatial restriction*, ignoring their origin and destination, i.e.,
$\vec{a_{1}}$⟶$\vec{a_{2}}$⟶⋯⟶$\vec{a_{T}}$⤏?

≈
≈
≈

?⤎$\vec{b_{T}}$⟶⋯⟶$\vec{b_{2}}$⟵$\vec{b_{1}}$
where:

$$d\left(\vec{a_{i}},\vec{b_{j}}\right)\leq \delta D,\;\forall \left(i,j\right)\;\;|\;\;2\leq i\leq T,\;j=T-\left(i-2\right),$$

where δD is a distance threshold.

In this case, we can pair stretches *i* and *j*, making a twofold assignment of spatial locations and times:

1. On one hand, we assign the location of the origin of each journey to the location of the destination of the other.
  $$\begin{array}{c} \vec{a_{1}}\;\to \;\vec{a_{2}}\;\to \cdots \to \;\vec{a_{T}}\;\to \;\vec{b_{1}}\\ \vec{b_{1}}\;\to \;\vec{b_{2}}\;\to \cdots \to \;\vec{b_{T}}\;\to \;\vec{a_{1}} \end{array}$$
2. On the other hand, we assign the travel time of the first trip of each journey to the travel time of the last trip of the other.
  $$\begin{array}{cc} tt\left(\vec{a_{T}}⟶\vec{b_{1}}\right) & =tt\left(\vec{b_{1}}⟶\vec{b_{2}}\right)\\ tt\left(\vec{b_{T}}⟶\vec{a_{1}}\right) & =tt\left(\vec{a_{1}}⟶\vec{a_{2}}\right) \end{array}$$

Therefore we obtain two complete journeys, both with a known destination.

Note that, in order to obtain journeys from stretches formed by the validations in $V_{k,d}$, $V_{k,d}$ must contain at least four validations. Consequently, the journeys this method derives are always *multi-modal*, thus including a change from one transport mode to another or from one line to another in the case of buses. In addition, validations in $V_{k,d}$ allow us to obtain *S* journeys at most, being *S* always an even number as journeys are determined using the corresponding *reverse pairs*.

In order to proceed to perform the pairing of incomplete journeys, our method uses two thresholds in time (δT) and space (δD). The search for journeys within the *S* stretches in $V_{k,d}$ will require a minimum of S/2 matches and a maximum of $C_{S,2}$. The algorithm presents a linear complexity with the number of validations. A detailed discussion on the selection process of these parameters can be found in Section 5.

## 4. Performance Evaluation Framework for Multi-Modal Public Transport Systems

The adapted trip chaining method generates the input data we need to construct a performance evaluation framework for multi-modal transport systems, which include a set of metrics that characterize the overall network and quantify its performance.

### 4.1. Dataset

In this study, we used data provided by the Consorcio Regional de Transportes de Madrid (CRTM), a public agency that coordinates and manages the public transport network in the Community of Madrid (8000 ${\mathrm{km}}^{2}$ and 6.6 million people). It is a wide network (11,000 km) that connects all 179 municipalities, including Madrid, Spain’s capital city. The dataset extends from 1 November 2018 to 28 February 2019 (4 months) and includes approximately 500 million transactions, widely exceeding other previous datasets such as those used in [5] (2 weeks and 95 million transactions) and [33] (1 month and 210 million transactions).

In 2017, the traditional magnetic ticket was substituted by a new contactless smart card. Nowadays, this is the only way of payment allowed in the public transport network. Under certain circumstances, it is permitted to use cash as the payment of single-trip tickets in urban buses and commuter trains. This form of payment accounts for a negligible percentage of trips, which is ultimately irrelevant for this study as it does not reflect multi-modal journeys. The smart cards can integrate up to three various modalities and can be validated on more than 33,000 stations. The card is only validated at the entry point of each transport mode.

Each validation includes: card ID (anonymized), time stamp, pay point (the boarding station, coded as a combination of the operator, the line, and the stop), the type of title (pass, multi-ticket, single ticket, tourist ticket… plus young ticket, senior ticket, children’s ticket…), and the type of discount (for large families, disabled persons…). The data were recorded with a temporal definition of 2 s. With the exception of buses, the SCD do not register the line and direction of the trip, just the station of validation.

The public transport network integrates six different modes: Subway (13 lines and 244 stations); Commuter Train (9 lines and 91 stations); Light Rail and Tram (4 lines and 58 stations); Urban Bus in Madrid Capital (216 lines and 10,563 stops); Intercity Bus (328 lines and 18,115 stops); and Urban Bus in the other Municipalities (52 lines and 4684 stops). Every line includes two directions. The bus fleet is equipped with an AVL technology, which allows us to obtain the line and stop directly from the SCD, with no additional processing.

This overall public transport network delivers high volumes of trips, registering validations that can exceed 5,000,000 a day. Among these, the multi-modal set of cards with four validations or more account for 59%.

Apart from the SCD, the CRTM provided us with a set of metadata that specifies the spatial location of every station in the network.

We applied the adapted trip chaining method to the multi-modal journeys in this extensive database, creating the dataset we used as the input for the performance evaluation framework we propose. The potential of this framework resides in, first, its data-driven nature and, second, its ability to provide detailed information for each specific day of the year. Next, we present the metrics that form the performance evaluation framework, particularized for 23 November 2018, as an illustrative example of the results.

### 4.2. Statistical Characterization

**Figure 2:**
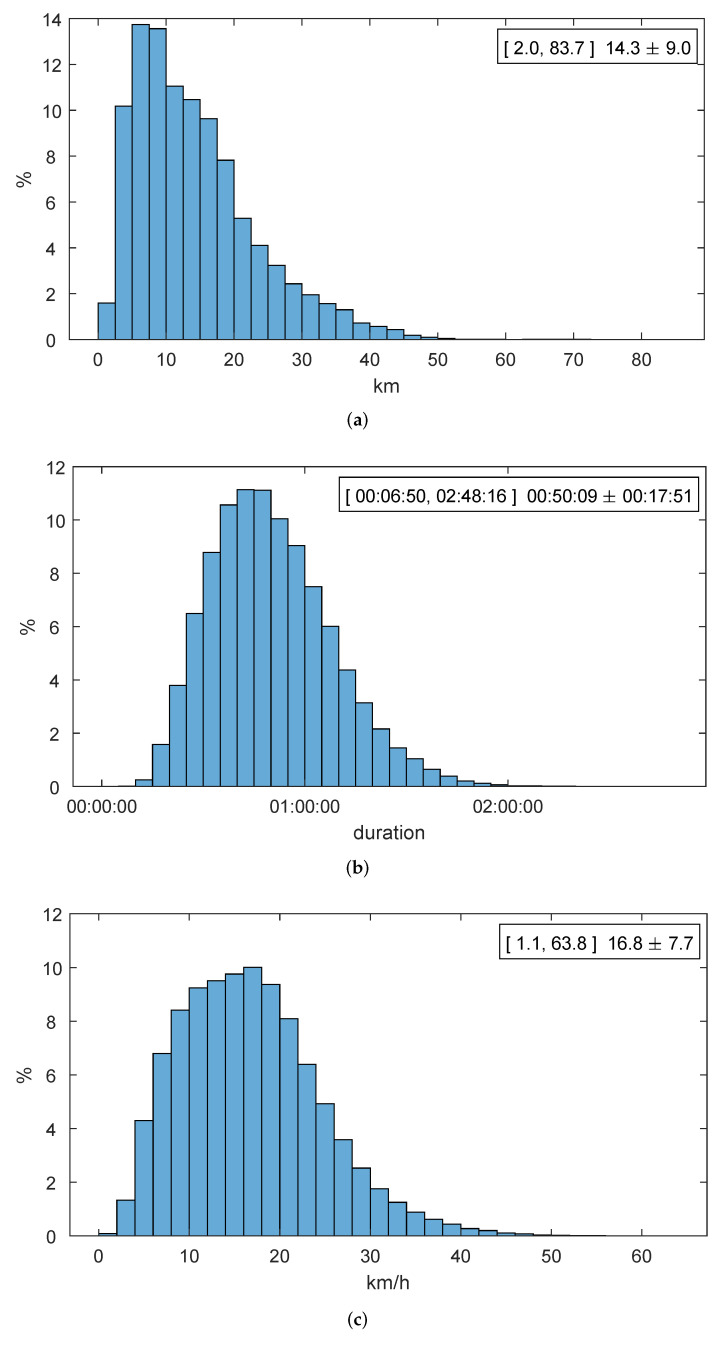
Statistical characterization for journeys (23 November 2018): (**a**) distance. (**b**) duration. (**c**) speed.

As the first basic metric to evaluate a public transport system, Figure 2 shows the statistical characterization of the reconstructed trips. On the top right corner of the histogram of each magnitude (distance, duration, and speed) we indicate its minimum, maximum, mean, and standard deviation.

The information presented in Figure 2 allows a direct interpretation of the public transport network in the Comunidad de Madrid: The average traveler traverses approximately 14km in a straight line to reach a place of work, study, or leisure, investing 50min. In both outbound and inbound journeys. Thus, this rider travels at an average speed of almost 17km/h.

### 4.3. Signature of a Public Transport System

**Figure 3:**
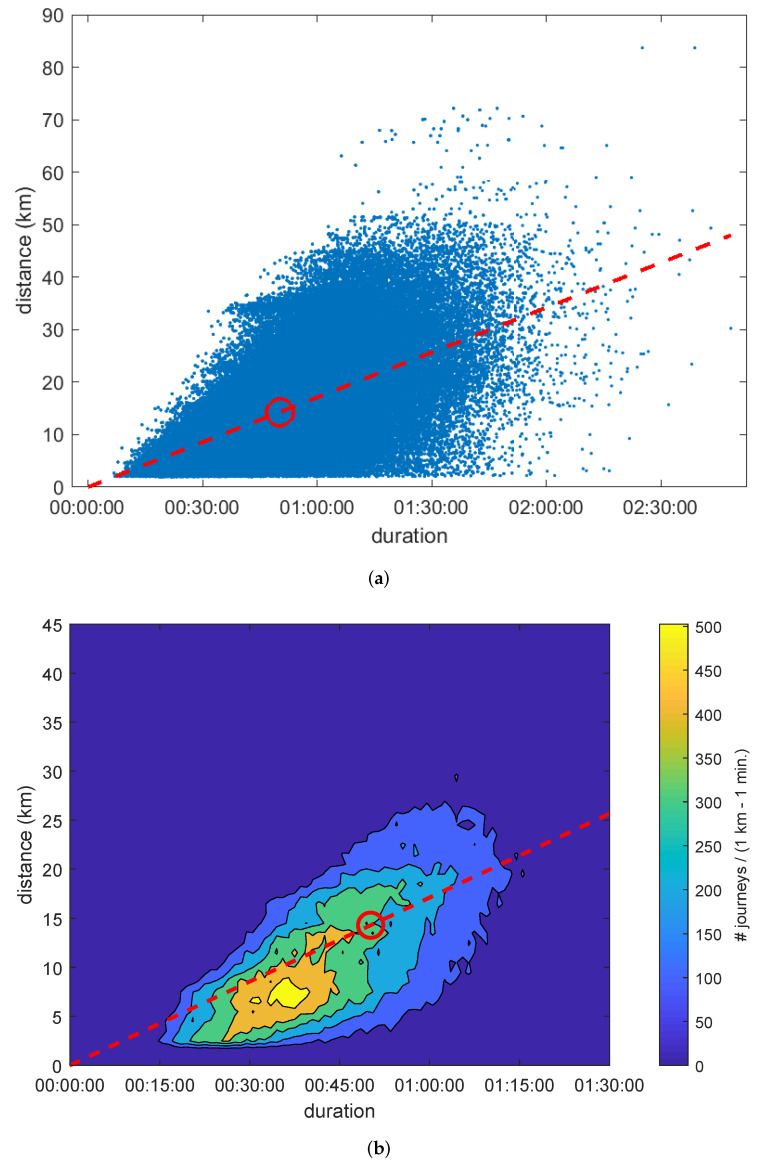
Space-time representation of journeys (23 November 2018): (**a**) points of individual journeys; (**b**) contour lines.

In Figure 3a, each blue point represents a journey with a specific duration (X axis) and traversed distance (Y axis). The red circle indicates the average journey and the slope of the discontinuous red line coincides with its velocity. This spatio-temporal representation allows us to characterize the performance of any public transport system. Every point underneath the line represents a journey that was *slower* than the average. In consequence, these points are able to highlight connections between geographical areas for which the network provides a poor transport solution. Thus, an optimal public transport system should aim for a high average velocity (represented by a steep slope) and gathering every journey around it so that all areas are optimally connected.

The high number of points shown in Figure 3a hides part of the information. Consequently, we present Figure 3b, which shows the contour lines for the bivariate histogram of journeys in bins of 1km×1min.; note the zoom applied to the axis. The area with a higher number of journeys beneath the discontinuous red line corresponds to the grid given by 37–38min. and 7–8km, showing a velocity of 12km/h (obviously lower than the speed of the average journey). We name this representation the *signature of a transport system* and provides a direct visualization of the spatio-temporal distribution of journeys. The signature of a transport system will reflect any future modifications made on the network (increasing the number of stations, restructuring the lines, extending the inter-modal transportation hubs, changing timetables, etc.), thus providing a tool to compare the results of these actions. Successful modifications will contribute to *slender* the shape of the contour lines, i.e., making them more elliptical and aligned to the line.

### 4.4. Journeys Departure Time

**Figure 4:**
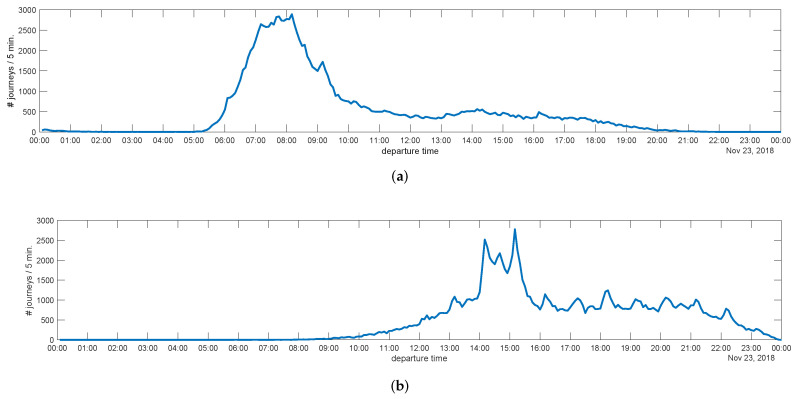
Number of journeys depending on the time of departure (23 November 2018): (**a**) outbound journeys; (**b**) return journeys.

Figure 4 shows the number of journeys versus the time of departure with an aggregation period of 5min. We can observe that most outbound journeys start in the morning, during a relatively narrow time interval (the great majority between 07:00 and 09:00). Return journeys mainly occur in the afternoon and present a much higher variability (from 13:00 to 22:00, with a significant peak between 14:00 and 16:00). This reflects the typical commuter behavior as reported in works such as [29].

### 4.5. Operating Speed

The CRTM uses the *operating speed* as a parameter to characterize each transport mode: 23km/h for subway, 47km/h for commuter train, etc.

**Figure 5:**
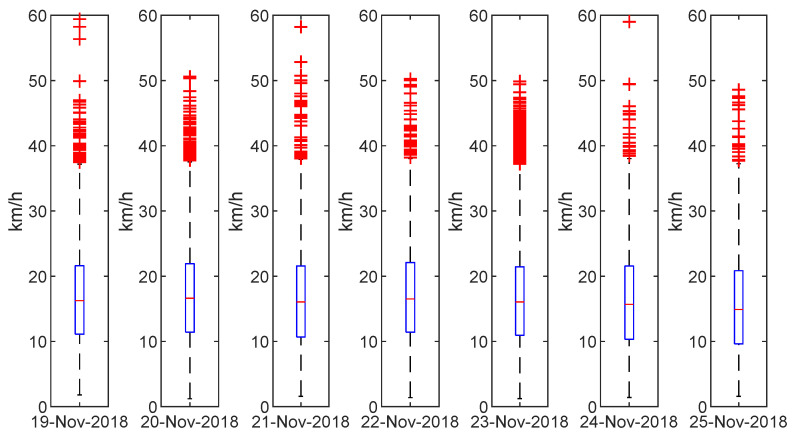
Boxplot of speed: week of 19 November 2018 (Mon) to 25 November 2018 (Sun).

The average velocity of all journeys reconstructed by the proposed method can be interpreted as the operating speed of the transport system as a whole. This way it represents the average velocity at which passengers travel in the transport network (including the travel, waiting, transfer times, and walking times). We can observe in Figure 5 the regularity of this variable during a complete week; note that it keeps this regularity even on weekends. Consequently, the operating speed can be considered an intrinsic parameter of an inter-modal public transport network, thus characterizing it. As such, it allows a simple and straightforward comparison of the efficiency of public transport networks within different cities and regions.

### 4.6. Enhanced OD Matrices

**Figure 6:**
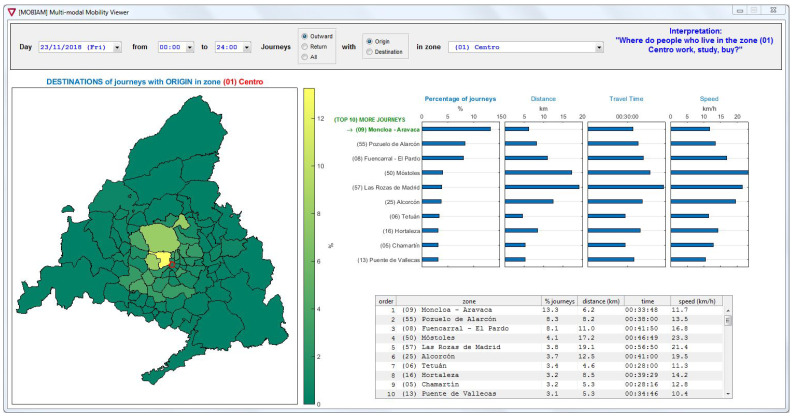
Screenshot of the software tool.

From the information about individual journeys, we can easily obtain the corresponding OD matrices. The concise mobility survey carried out by CRTM in 2014 divided the territory in 84 zones. We have used this same spatial aggregation to construct the resulting OD matrices in this study. Figure 6 shows a screenshot of the software tool we have developed to obtain reconstructed journeys and OD matrices. The tool was developed in Matlab and aims at providing meaningful information for the assessment of multi-modal public transport systems. It is fed with data corresponding to paired journeys, which result from the adapted trip chaining method. The tool provides control buttons to select a specific date and time interval; in addition, we can choose the analysis to be performed on the outbound journeys, the return journeys, or both; finally, we can define each zone as the origin or destination of all the journeys. Thus, each setting of the control parameters provides a row or a column of the complete OD matrix resulting from the journeys selected by the applied filters. The information is presented in different modes: as colors on a map, values in a table, and bar charts; each of them can be reordered depending on a set of magnitudes: percentage of journeys (i.e., the values in the OD matrix), distances, travel times, and speeds.

As a consequence, this tool generates *enhanced* OD matrices, extending the basic information about the distribution of journeys throughout the network, with the distance, travel time, and speed corresponding to each OD pair. As an example, Figure 6 shows that zone “(09) Moncloa–Aravaca” is the destination of 13.3% of the outbound journeys originating in zone “(01) Centro”, elementary OD matrix information. On top of it, we also know that these journeys imply an average traveled distance of 6.2km and an average travel time of 00:33:48, resulting in an average speed of 11.7km/h. Furthermore, we can observe that the zone with the best connectivity is “(50) Móstoles”, which ranks first in terms of operating speed (23.3km/h) and forth regarding the number of journeys.

## 5. Discussion, Conclusions, and Further Research

### 5.1. Comparative Study

Characterizing mobility often encompasses an inherent problem for validation: the lack of a solid ground truth [34]. For this reason, we must rely on comparative approaches that may highlight an underlying common structure.

In this respect, let us first perform a comparative analysis of the results we obtained and those reflected in the latest Household Mobility Survey performed in the Comunidad de Madrid. This survey was carried out in 2018 and included 85,064 transport users throughout the complete region. In order to compare our results to this survey, we segmented the overall OD matrices considering the transport modes employed. Table 1 shows the percentages of trips traveled in any combination of two transport modes in the network. This set of trips is the largest, accounting for more than 90% of the total mobility. Columns **HMS18** and **PEF** include the corresponding percentages of each category resulting from the Household Mobility Survey and the performance evaluation framework, respectively. We can observe how both approaches reach very similar results in each category (deviations below 7.5%), which indicates the capability of the proposed framework to represent the detailed mobility in the Comunidad de Madrid.

In addition, let us visit again the histograms presented in Figure 2 to observe that they show an outstanding regularity and resemble the usual patterns observed in public transport systems, such as those reported in [35], which were exactly reconstructed from exit data captured in Shanghai’s (China) subway. Apart from their regularity, these histograms are also consistent, i.e., the definite shape of the distribution is shown with approximately the first 5000 completed journeys. This fact indicates that our performance evaluation framework precisely represents the multi-modal mobility in public transport systems.

### 5.2. Parameter Selection

The adapted trip chaining method includes the selection of two thresholds in time, δT, and distance, δD. These parameters are specific to the public transport system under study; thus, we have to determine them accordingly. In this subsection, we will discuss the impact of the selection of these parameters.

Let us remember that δT fixes the maximum time interval accepted between two consecutive validations to be considered part of the same journey. If we choose a low value of δT, the method will not include journeys that comprise long trips. The duration of these trips depends on the specific transport network. As an example, intercity buses in the Comunidad de Madrid connect peripheral areas with Madrid’s city center, taking 50 to 55 min to be completed. Thus δT must be adapted to this duration. On the other hand, if we take an excessive value, the method will group together trips belonging to different journeys, which will not be paired correctly. This will slightly reduce the number of detected journeys. Considering these two limitations, we carried out a sensitivity analysis that resulted in selecting δT=1h (see Table 2).

Similarly, the distance threshold δD is determined by the network’s topology. It represents the typical distance between stations of different modes. In this respect, we must consider distances in transport hubs (including intercity buses, subway, and commuter trains), stations that comprise more than one transport mode (mainly subway and commuter train), and streets. Note that in this case the number of journeys will grow with increasing values of δD at the expense of reducing their spatial definition. Consequently, we must choose a δD that results in a relatively stable number of journeys. Following this rule, we selected δD=200m for the public transport network in the Comunidad de Madrid (see Table 3).

### 5.3. Conclusions and Further Research

Multi-modal mobility represents a considerable percentage of the overall mobility in a city or region. Thus, it is the key to developing tools explicitly tailored to assess how public transport networks respond to it. To this end, we have created a framework for the evaluation of multi-modal public transport systems that uses a data-driven approach based on journeys reconstructed by means of an adapted trip chaining method.

The performance evaluation framework we have proposed opens up a wide range of possibilities for further research. In order to completely characterize mobility, a new set of aspects must be taken into account. First, unimodal and sporadic journeys in public transport networks must be analyzed. Second, mobility on private vehicles, including cars, motorbikes, bikes, scooters, etc., must be incorporated to the model. Finally, pedestrian flows and the emerging shared mobility have to be addressed.

The next step of our investigation will incorporate the share of unimodal recurrent mobility, i.e., journeys using a single mode, to the input dataset to our performance evaluation framework. The required assignment of destinations is a priori feasible, using SCD from a set of dates. The problem is determining the travel times for the last trip. In this respect, we will consider specific data of the service and averages derived from other users’ journeys. In any case, this is a complex process that will require further research and the development of specific algorithms.

The accurate assessment of multi-modal mobility is important for the future redesigning of public transport systems. This includes, for example, the construction of direct links that avoid transfers and improve the overall urban mobility. Using a data-driven approach enables the evaluation of the transport system 365 days a year, which allows the extraction of meaningful knowledge about passenger behavior, its evolution, and their response to new changes and updates in the network and services.

## Figures and Tables

**Table 1:** Household Mobility Survey and Reverse Pairing Method.

	HMS18	PEF
Intercity Bus–Subway	24.38%	29.82%
Commuter Train–Subway	23.88%	31.17%
Urban Bus–Subway	21.89%	20.97%
Urban Bus–Urban Bus	15.92%	8.72%
Commuter Train–Urban Bus	5.47%	4.69%
Urban Bus–Intercity Bus	5.47%	3.05%
Intercity Bus–Intercity Bus	2.99%	1.59%

**Table 2:** Sensitivity Analysis of the Time Threshold δT.

Parameter Value	45min.	60min.	90min.
%Journeys	↓11.37%	100%	↓1.79%

with δD=200m.

**Table 3:** Sensitivity Analysis of the Distance Threshold δD.

Parameter Value	175m	200m	225m
% Journeys	↓1.75%	100%	↑1.31%

with δT=1h.

## Data Availability

The data presented in this study are available on request from Consorcio Regional de Transportes de Madrid. The data are not publicly available due to its proprietary nature.
